# A Case Report of Navigating Dual Respiratory Challenges: From Pulmonary Tuberculosis (PTB) to Allergic Bronchopulmonary Aspergillosis (ABPA)

**DOI:** 10.7759/cureus.64735

**Published:** 2024-07-17

**Authors:** Bana Mary Manishaa, Jereen Varghese, Nithin Thomas

**Affiliations:** 1 Pulmonology Department, Shri Sathya Sai Medical College and Research Institute, Chennai, IND

**Keywords:** anti-tubercular therapy (att), allergic bronchopulmonary aspergillosis (abpa), aspergillus fumigatus, th2 response, th1 response, corticosteroids, delayed diagnosis, pulmonary tuberculosis (ptb)

## Abstract

This case study describes a unique scenario in which allergic bronchopulmonary aspergillosis (ABPA) was identified following treatment for pulmonary tuberculosis (PTB). ABPA is a complex pulmonary disorder that is often overlooked due to its nonspecific clinical presentation, especially in individuals concurrently diagnosed with tuberculosis (TB). Despite initial TB diagnosis and treatment, a 28-year-old male continued to experience respiratory symptoms, prompting further investigation that revealed underlying ABPA. This case underscores the importance of emphasizing the critical role of maintaining a high level of suspicion for ABPA in TB patients with persistent symptoms, highlighting the need for timely recognition and management to minimize further lung damage and improve patient outcomes.

## Introduction

Allergic bronchopulmonary aspergillosis (ABPA) is a complex pulmonary disorder that is often underdiagnosed due to its nonspecific clinical presentation and similarities to other respiratory conditions, such as tuberculosis (TB) [1]. TB can sometimes obscure underlying ABPA, leading to delayed diagnosis and inappropriate treatment [2]. ABPA is characterized as a hypersensitive response to the fungus *Aspergillus fumigatus,* which triggers a chronic inflammatory response in the lungs, often observed in individuals with preexisting asthma or cystic fibrosis [3]. Although relatively uncommon, ABPA can cause substantial morbidity and mortality if not addressed promptly [3]. Diagnosing ABPA can pose challenges due to its nonspecific clinical presentation, yet it generally requires a comprehensive approach combining serological testing for *Aspergillus*-specific IgE antibodies, serum total IgE, *Aspergillus*-specific IgG antibodies, absolute eosinophil count, and imaging consistent with ABPA according to the revised International Society for Human and Animal Mycology (ISHAM)-ABPA working group [4]. Treatment for ABPA typically involves oral prednisolone or itraconazole, but the optimal management approach is still a matter of debate [4].

## Case presentation

**Figure 1:**
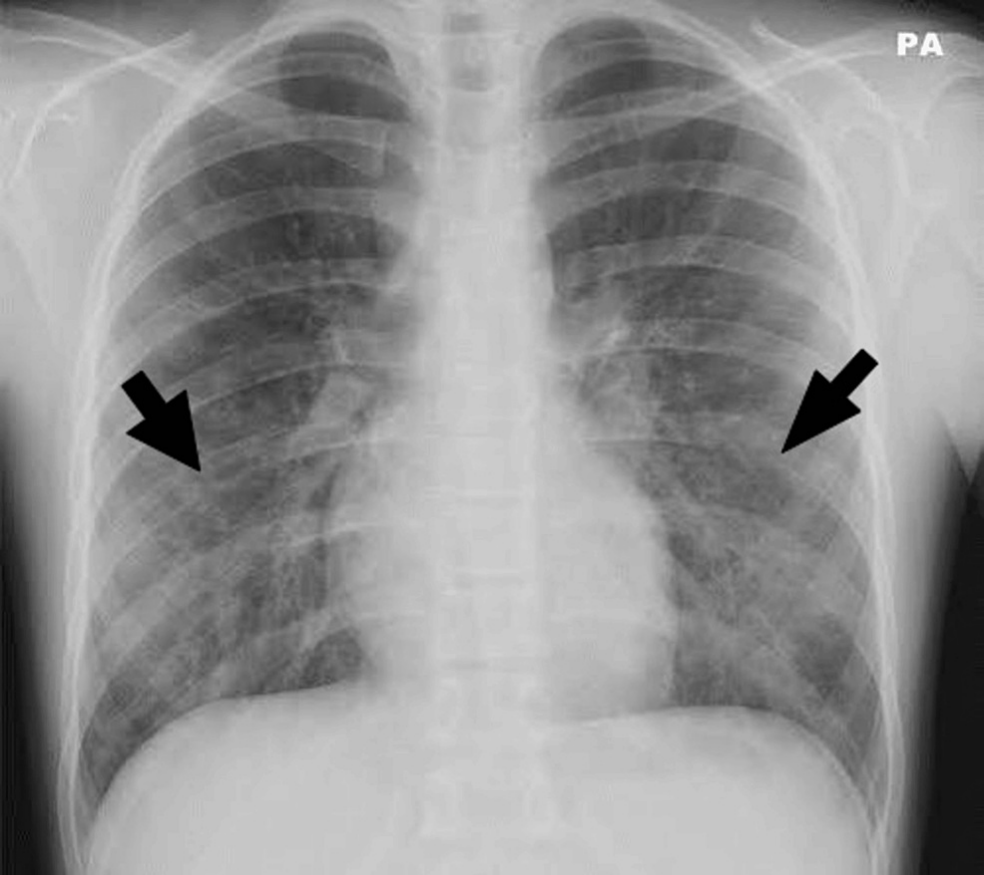
Chest X-ray postero-anterior (PA) view revealed non-homogenous opacities in the bilateral mid and lower zones.

A 28-year-old male presented with symptoms of cough with mucoid expectoration, breathlessness of Grade 3 mMRC (modified Medical Research Council) with wheezing, fever with chills and rigors, and loss of appetite, which had been worsening over the past two months. He had a history of recurrent sneezing and rhinorrhea but no history of food/drug allergy or atopic dermatitis. He was diagnosed with asthma through spirometry and has since been on a combination of inhaled corticosteroids (ICSs) and long-acting beta-2 agonists (LABAs) for therapy. On examination, he was moderately built with stable vitals. Auscultation revealed normal breath sounds with coarse crackles in bilateral inter-scapular, infra-scapular, and infra-axillary areas. Expiratory wheezing was heard all over the lung fields. Blood examination revealed a normal total leukocyte count, an elevated erythrocyte sedimentation rate (ESR) of 90 mm/hour, and an elevated absolute eosinophil count (AEC) of 1200 cells/mm^3^. The chest X-ray postero-anterior (PA) view revealed non-homogenous opacities in the bilateral mid and lower zones (Figure 1). Sputum examination for gram stain was negative, bacterial culture was sterile, acid-fast bacilli (AFB) 2+ was present, and GeneXpert detected rifampicin-sensitive *Mycobacterium tuberculosis*. Hence, he was started on an ATT (fixed-dose combination) intensive phase. 

**Figure 2:**
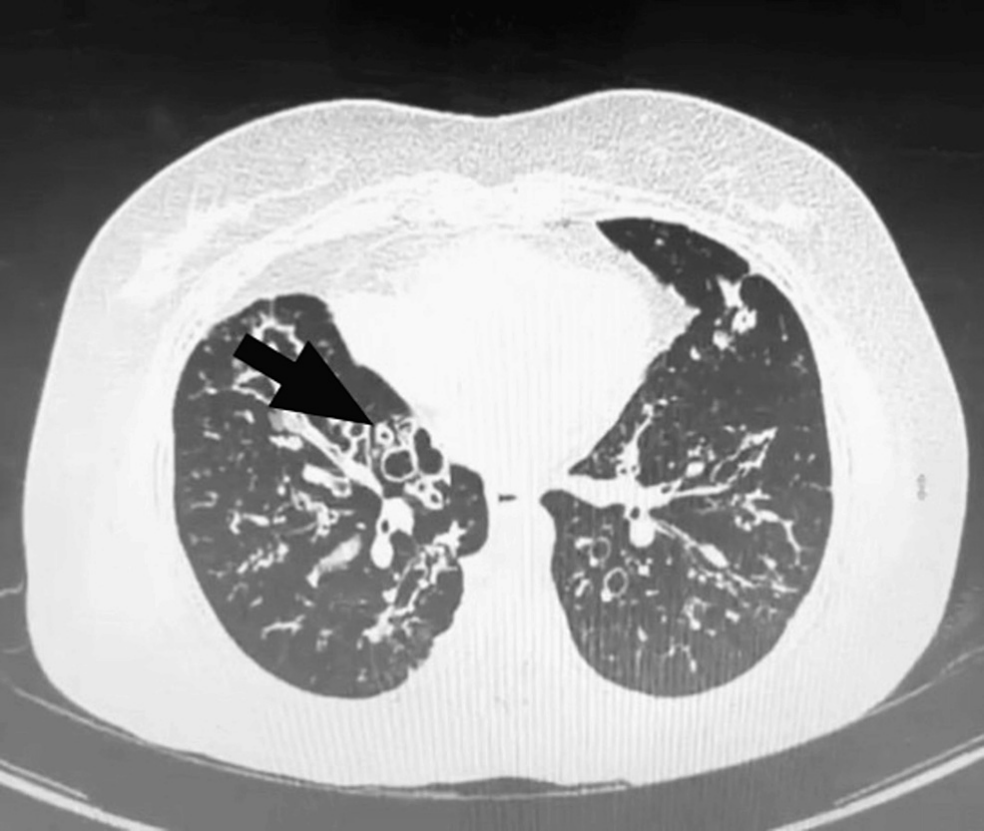
Plain high-resolution CT thorax revealed varicose and cystic bronchiectasis changes in the bilateral middle and lower lobes with bronchial wall thickening, scattered centrilobular nodular, and tree-in-bud opacities with scattered areas of patchy opacities.

Despite being on ATT for two months, the patient exhibited worsening cough and breathlessness. However, drug resistance to mycobacteria was ruled out as the MTB culture was negative and the fever subsided. Due to persistent symptoms, a history of asthma, and hyper-eosinophilia, the patient was further evaluated for ABPA. Plain high-resolution CT thorax revealed varicose and cystic bronchiectasis changes in the bilateral middle and lower lobes with bronchial wall thickening, scattered centrilobular nodular, and tree-in-bud opacities with scattered areas of patchy opacities (Figure 2). Further evaluation showed elevated total serum IgE levels (1,979 IU/mL), positive serum-specific IgE against *Aspergillus fumigatus* (0.84 kUA/L), positive skin prick test with Aspergillin, and positive IgG against *Aspergillus fumigatus* (35 mg/L). Spirometry revealed a reduced FEV1/FVC ratio of 0.60 and a reduced FEV1 of 65% predicted, with significant improvement in FEV1 of 15% predicted post-bronchodilator. Therefore, the patient was diagnosed as a new case of microbiologically confirmed pulmonary TB masking underlying ABPA. The patient was advised to continue the ATT regimen and to use the metered-dose inhaler (MDI) with a spacer for the underlying obstructive airflow limitation, along with oral prednisolone. During monthly follow-ups, the patient has shown significant clinical and radiological improvement.

## Discussion

Concomitant ABPA with active pulmonary tuberculosis (PTB) represents an atypical presentation that has been infrequently documented in the medical literature [5]. Notably, a significant proportion of ABPA patients may have been initiated on ATT prior to the diagnosis of ABPA [6]. Research indicates that the Th1 response to *Mycobacterium tuberculosis* infection acts to suppress the Th2 response and reduces the risk of allergy and asthma development [7]. However, following ATT, the Th1 response diminishes, resulting in a relative predominance of the Th2 response, thereby increasing the risk of asthma exacerbations and ABPA. The treatment of ABPA aims to manage acute inflammatory episodes and curtail progressive lung injury. The primary therapeutic approach encompasses systemic glucocorticoids in conjunction with measures to minimize *Aspergillus* exposure to prevent relapse following steroid discontinuation [8]. Vigilant monitoring of the response to steroids is essential after eight to 12 weeks of initiating therapy, involving a combination of clinical, immunological, and imaging findings for ABPA [4]. Oral prednisolone may be continued until a favorable clinical or radiologic response is observed and a substantial decline in IgE levels is achieved[9]. In conclusion, it is imperative to consider ABPA in patients with active PTB who exhibit poor response to ATT or suffer from recurrent pulmonary symptoms. Timely recognition and management of ABPA can avert further lung damage and enhance patient outcomes.

## Conclusions

This case report presents an intriguing instance of the coexistence of ABPA with active PTB. Considering the potential for adverse outcomes and compromised lung function associated with both ABPA and TB, it is essential to maintain a heightened level of suspicion for ABPA in asthmatic individuals experiencing persistent respiratory symptoms during or after ATT for PTB. This vigilance ensures timely identification and proper management of ABPA.
